# Prognostic and Treatment-Associated Survival Effects of Microsatellite Instability Across Disease Stages in Colon Cancer: An NCDB Analysis

**DOI:** 10.1007/s12029-026-01493-z

**Published:** 2026-05-20

**Authors:** Mohyeddine El Sayed, Sassine Youssef, Maha Chaccour, Melhem El Harati

**Affiliations:** 1 Hollings Cancer Center, Medical University of South Carolina, Charleston, SC USA; 2 Department of Pulmonary and Critical Care, Medical University of South Carolina, Charleston, SC USA

**Keywords:** Microsatellite instability status, Colon cancer, Prognosis, Treatment modalities

## Abstract

**Background:**

Microsatellite instability (MSI) is an established molecular biomarker in colorectal cancer (CRC). However, its stage-specific prognostic value and treatment-modifying role in colon cancer remain incompletely defined, particularly across contemporary multimodality treatment strategies.

**Methods:**

We conducted a retrospective cohort study using the National Cancer Database, including patients aged ≥ 20 years diagnosed with colon cancer between 2004 and 2020 with documented tumor stage and MSI status. MSI was categorized as microsatellite stable (MSS), MSI-low (MSI-L), or MSI-high (MSI-H). Multivariable Cox proportional hazards regression models were used to evaluate the association between MSI status and overall survival (OS), stratified by disease stage and treatment combinations. Patients who died within 90 days of diagnosis were excluded to minimize immortal-time bias.

**Results:**

A total of 129,484 patients met inclusion criteria. MSI-H tumors comprised 16.8% of cases. The prognostic impact of MSI status varied by stage. MSI-H was not associated with OS in stage 0–I disease but conferred a significant survival advantage in stage II (HR 0.86, *P* < 0.001), stage III (HR 0.71, *P* < 0.001), and stage IV disease (HR 0.65, *P* < 0.001). MSI-L tumors demonstrated no consistent survival benefit, with a modest association observed only in stage IV (HR 0.89, *P* = 0.015). Treatment-stratified analyses revealed that in stage II disease, MSI-H was associated with improved survival only among patients treated with surgery alone (HR 0.83, *P* < 0.001). In contrast, MSI-H tumors demonstrated substantial survival benefit across multiple treatment pathways in stage III and IV disease, including surgery plus chemotherapy (stage III HR 0.72; stage IV HR 0.70) and immunotherapy-containing regimens (stage III HR 0.28; stage IV HR 0.47; all *P* < 0.01).

**Conclusion:**

MSI status demonstrates a stage-dependent role in colon cancer, serving as a prognostic biomarker in early-stage disease and a predictive marker for immunotherapy-associated survival benefit in advanced stages.

**Supplementary Information:**

The online version contains supplementary material available at 10.1007/s12029-026-01493-z.

## Introduction

In 2022, colorectal cancer (CRC) was the third most common malignancy and the second leading cause of cancer-related mortality worldwide, with 1.9 million new cases and 900,000 deaths [1]. Colorectal carcinogenesis is driven predominantly by two distinct pathways of genomic instability: chromosomal instability, present in approximately 85% of cases, and microsatellite instability (MSI), which occurs in about 15% of tumors [2].

MSI arises from a deficiency of the DNA mismatch repair (MMR) system, which corrects DNA mutations [3]. In CRC, MSI tumors can be sporadic (80%), often due to hypermethylation of the MLH1 gene promoter, or hereditary (20%), as seen in Lynch Syndrome which is caused by germline mutations in one of the MMR genes [2]. MSI is assessed using a standardized panel of microsatellite markers, classifying tumors as MSI-high (MSI-H) when instability is detected in two or more loci, or MSI-low (MSI-L) when only a single marker is affected [4].

Beyond traditional Tumor–Node–Metastasis (TNM) staging, MSI status has emerged as an important molecular biomarker in CRC [5–7]. Unlike the poor prognosis associated with KRAS mutations, MSI tumors have consistently been linked to improved survival despite exhibiting high-risk pathological features, including poor differentiation, larger tumor size, and lymphocytic infiltration [8–11]. Furthermore, MSI status also carries predictive implications, as MSI-H tumors derive limited benefit from adjuvant chemotherapy in stage II disease while demonstrating marked sensitivity to immune checkpoint inhibition in advanced settings [12, 13].

Despite this growing body of evidence, the prognostic and predictive significance of MSI across individual disease stages and treatment strategies remains incompletely defined, particularly in colon cancer analyzed independently of rectal disease. Moreover, real-world data examining how MSI status modifies survival outcomes across multimodality treatment combinations are limited. Therefore, the objective of this study was to evaluate the stage-specific prognostic impact of MSI status in colon cancer and to assess its treatment-modifying effect on overall survival across commonly utilized therapeutic approaches.

## Methods

This retrospective cohort study utilized data from the National Cancer Database (NCDB) PUF 2021, a comprehensive national clinical oncology database that includes information on patient demographics, tumor characteristics, treatments, and outcomes. Sponsored by the American College of Surgeons and the American Cancer Society, the NCDB gathers data from over 1,500 cancer centers across the United States [14]. This study was exempt from Institutional Review Board approval because the data were deidentified.

### Study Population

Patients aged ≥ 20 years with a diagnosis of colon cancer between 2004 and 2020 with documented tumor stage and microsatellite instability (MSI) status were eligible for inclusion. Exclusion criteria included multiple primary malignancies, unspecified tumor grade, missing race or ethnicity data, incomplete survival information, unknown metastatic status, or incomplete treatment data. MSI status was categorized as microsatellite stable (MSS), MSI-low (MSI-L), or MSI-high (MSI-H), according to NCDB definitions.

### Variables

Demographic variables analyzed included sex, age, race, and ethnicity. Tumor-related variables encompassed primary tumor site, tumor size, tumor grade, and Charlson-Deyo comorbidity score. Treatment variables included surgery, chemotherapy, immunotherapy, and radiation.

### Statistical Analysis

Categorical variables were summarized as frequencies and percentages and compared using the Pearson chi-square test. The association between MSI status and OS was evaluated using multivariable Cox proportional hazards regression models stratified by disease stage, with hazard ratios (HRs) and 95% confidence intervals (CIs) reported. Additional multivariable analyses examined the effect of MSI status on OS across treatment combinations. Due to limited sample size, regression analyses were not performed for some treatment combination. To minimize immortal-time bias, patients who died within 90 days of diagnosis were excluded. Treatment variables (chemotherapy, radiation, immunotherapy, surgery) were modeled as baseline covariates as per standard NCDB methodology. Variables included in multivariable models were selected based on clinical relevance and statistical significance in univariable analyses. All statistical analyses were conducted using IBM SPSS Statistics, version 30.0 (IBM Corp., Armonk, NY, USA). A two-sided p value < 0.05 was considered statistically significant.

##  Results

**Fig. 1:**
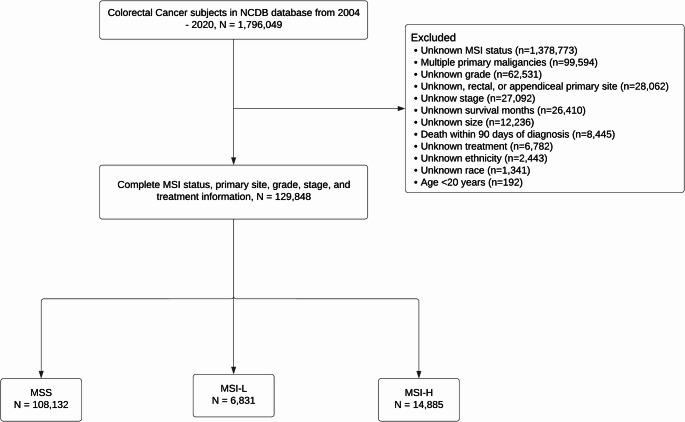
CONSORT diagram of included and excluded patients

The initial cohort consisted of 1,796,049 CRC patients diagnosed between 2004 and 2020. Out of these, 129,484 patients met the study’s inclusion and exclusion criteria (Fig. 1). MSI-H tumors were more common in females, White patients, and older adults, and showed higher comorbidity scores. They demonstrated a distinct tumor profile, with a strong predilection for the right colon, poorer differentiation, and larger tumor size. MSI-H cancers were also characterized by earlier-stage presentation. MSI-H patients were less likely to receive chemotherapy or radiation. Table 1 presents a comparison of baseline characteristics across different cohorts.

**Table 1:** Patient demographics and clinical characteristics, stratified by MSI status

*N* = 129,848				
	MSS *N* = 108,132	MSI–L *N* = 6,831	MSI–H *N* = 14,885	*P*-value
**Demographics**				
Sex				
Male	52.0	47.5	40.9	< 0.001
Female	48.0	52.5	59.1	
Age group, years				
20–49	15.1	13.8	12.5	< 0.001
≥50	84.9	86.2	87.5	
Race				
Whites	80.4	81.0	87.0	< 0.001
Black	13.1	11.9	8.4	
Other	6.5	7.1	4.6	
Ethnicity				
Non-Hispanic	92.8	91.6	93.4	< 0.001
Hispanic	7.2	8.4	6.6	
Charlson-Deyo Score				
Score 0	70.4	71.3	66.2	< 0.001
Score 1	18.6	19.2	19.1	
Score 2	6.0	4.9	7.8	
Score 3+	5.0	4.6	6.9	
**Tumor characteristics**				
Primary site				
Right Colon	51.1	59.3	81.5	< 0.001
Left Colon	38.7	32.4	16.4	
Rectosigmoid Junction	10.3	8.3	2.1	
Grade				
Well-differentiated	10.6	9.8	7.4	< 0.001
Moderately differentiated	74.7	70.4	57.7	
Poorly differentiated	13.0	17.9	31.8	
Undifferentiated	1.7	1.9	3.1	
Size, cm				
< 3.0	22.9	20.4	15.6	< 0.001
3.0–4.9	36.3	34.5	25.8	
>5.0	40.8	45.1	58.6	
Stage				
0	0.7	0.6	0.3	< 0.001
I	18.8	17.8	19.5	
II	31.8	34.7	44.6	
III	35.5	35.7	29.4	
IV	13.2	11.2	6.2	
**Treatments**				
Surgery	100	100	100	-
Chemotherapy	48.0	43.9	31.6	< 0.001
Immunotherapy	5.4	4.1	3.8	< 0.001
Radiation	2.6	2.6	1.2	< 0.001

When stratified by disease stage, the prognostic effect of MSI status varied substantially across stages. In stage 0 and stage I disease, neither MSI-L nor MSI-H was associated with a significant difference in overall survival compared with MSS tumors. In stage II, MSI-H tumors demonstrated a significant survival advantage (HR 0.86, *P* < 0.001), whereas MSI-L showed no association with outcomes. This survival benefit of MSI-H persisted and became more pronounced in stage III (HR 0.71, *P* < 0.001), while MSI-L tumors demonstrated no survival benefit. In stage IV disease, MSI-L tumors showed modest survival benefit (HR 0.89, *P* = 0.015), while MSI-H tumors had the greatest survival advantage (HR 0.65, *P* < 0.001) (Table 2).

**Table 2:** Overall survival by MSI Status across disease stages in colon cancer

	HR	95% CI	*P* value
**Stage 0**			
MSS	Referent	-	-
MSI-L	0.69	0.22, 2.22	0.538
MSI-H	1.96	0.83, 4.65	0.127
**Stage I**			
MSS	Referent	-	-
MSI-L	1.08	0.93, 1.24	0.308
MSI-H	1.09	0.98, 1.22	0.126
**Stage II**			
MSS	Referent	-	-
MSI-L	1.00	0.91, 1.09	0.914
MSI-H	0.86	0.81, 0.92	**< 0.001**
**Stage III**			
MSS	Referent	-	-
MSI-L	0.93	0.87, 1.01	0.072
MSI-H	0.71	0.66, 0.75	**< 0.001**
**Stage IV**			
MSS	Referent	-	-
MSI-L	0.89	0.82, 0.98	**0.015**
MSI-H	0.65	0.59, 0.71	**< 0.001**

Age-stratified analyses demonstrated that the association between MSI status and overall survival was largely consistent across age groups (Supplementary Table 1). In stage III disease, MSI-H was associated with improved survival in both younger (HR 0.52, *P* < 0.001) and older patients (HR 0.73, *P* < 0.001). Similarly, in stage IV disease, MSI-H conferred a survival benefit in both younger (HR 0.70, *P* < 0.001) and older patients (HR 0.63, *P* < 0.001). In earlier stages, the effect of MSI-H was less pronounced and not consistently statistically significant among younger patients.

Across treatment combinations and stages, the prognostic effect of MSI status varied considerably. In stage I, neither MSI-L nor MSI-H did demonstrate a significant survival difference across any treatment pathway. In stage II, MSI-H was associated with improved survival only among patients treated with surgery alone (HR 0.83, *P* < 0.001), whereas MSI-L was associated with worse survival among patients treated with surgery and chemoradiation (HR 1.72, *P* = 0.046). In stage III, MSI-H consistently conferred a survival benefit across multiple treatment pathways, including surgery only (HR 0.77, *P* < 0.001), surgery plus chemotherapy (HR 0.72, *P* < 0.001), and surgery plus chemotherapy with immunotherapy (HR 0.28, *P* < 0.001). Whereas MSI-L showed no prognostic effect across any treatment pathway. In stage IV disease, MSI-H conferred a significant survival advantage across several major treatment strategies, including surgery alone (HR 0.76, *P* = 0.011), surgery with chemotherapy (HR 0.70, *P* < 0.001), surgery with immunotherapy (HR 0.47, *P* = 0.003), and surgery with both chemotherapy and immunotherapy (HR 0.67, *P* < 0.001). In contrast, MSI-L demonstrated survival benefit among patients undergoing surgery alone (HR 0.76, *P* = 0.014) (Table 3).

**Table 3:** Overall survival by MSI Status across disease stages and treatment combinations in colon cancer

Treatment Combinations	HR	95% CI	*P* value	Count (%)
**Stage I**				
Surgery only				
MSS	Referent	-	-	20,015 (83.0)
MSI-L	1.08	0.94, 1.25	0.286	1,198 (5.0)
MSI-H	1.10	0.98, 1.23	0.110	2,886 (12.0)
Surgery & Chemotherapy				
MSS	Referent	-	-	164 (89.1)
MSI-L	1.59	0.35, 7.14	0.545	8 (4.4)
MSI-H	0.61	0.08, 4.61	0.635	12 (6.5)
Surgery & Chemotherapy & Radiation				
MSS	Referent	-	-	127 (89.4)
MSI-L	0.60	0.08, 4.50	0.619	9 (6.3)
MSI-H	NA	NA	NA	6 (4.3)
**Stage II**				
Surgery only				
MSS	Referent	-	-	26,185 (77.4)
MSI-L	0.98	0.89, 1.08	0.743	1,908 (5.6)
MSI-H	0.83	0.77, 0.90	**< 0.001**	5,743 (17.0)
Surgery & Chemotherapy				
MSS	Referent	-	-	7,198 (85.6)
MSI-L	1.00	0.76, 1.31	0.991	410 (4.9)
MSI-H	1.09	0.89, 1.35	0.395	795 (9.5)
Surgery & Radiation				
MSS	Referent	-	-	45 (77.6)
MSI-L	0.81	0.25, 2.63	0.722	7 (12.1)
MSI-H	0.62	0.19, 2.03	0.431	6 (10.3)
Surgery & Chemotherapy & Immunotherapy				
MSS	Referent	-	-	105 (85.4)
MSI-L	0.42	0.05, 3.36	0.414	2 (1.6)
MSI-H	0.48	0.15, 1.57	0.224	16 (13.0)
Surgery & Chemotherapy & Radiation				
MSS	Referent	-	-	780 (87.3)
MSI-L	1.72	1.01, 2.93	**0.046**	41 (4.6)
MSI-H	1.34	0.80, 2.23	0.264	72 (8.1)
**Stage III**				
Surgery only				
MSS	Referent	-	-	7,298 (80.5)
MSI-L	0.90	0.87, 1.12	0.869	536 (5.9)
MSI-H	0.77	0.70, 0.85	**< 0.001**	1,232 (13.6)
Surgery & Chemotherapy				
MSS	Referent	-	-	29,220 (86.2)
MSI-L	0.92	0.83, 1.02	0.100	1,761 (5.2)
MSI-H	0.72	0.66, 0.78	**< 0.001**	2,927 (8.6)
Surgery & Immunotherapy				
MSS	Referent	-	-	29 (42.6)
MSI-L	0.43	0.09, 2.03	0.285	7 (10.3)
MSI-H	1.06	0.34, 3.33	0.917	32 (47.1)
Surgery & Chemotherapy & Immunotherapy				
MSS	Referent	-	-	611 (77.1)
MSI-L	0.71	0.44, 1.14	0.159	48 (6.1)
MSI-H	0.28	0.18, 0.44	**< 0.001**	133 (16.8)
Surgery & Chemotherapy & Radiation				
MSS	Referent	-	-	1,205 (90.4)
MSI-L	1.08	0.73, 1.59	0.705	84 (6.3)
MSI-H	0.89	0.50, 1.58	0.691	44 (3.3)
**Stage IV**				
Surgery only				
MSS	Referent	-	-	1,817 (86.9)
MSI-L	0.76	0.61, 0.95	**0.014**	125 (6.0)
MSI-H	0.76	0.62, 0.94	**0.011**	149 (7.1)
Surgery & Chemotherapy				
MSS	Referent	-	-	7,112 (90.1)
MSI-L	0.98	0.86, 1.11	0.730	401 (5.1)
MSI-H	0.70	0.61, 0.80	**< 0.001**	381 (4.8)
Surgery & Immunotherapy				
MSS	Referent	-	-	65 (42.8)
MSI-L	0.43	0.10, 1.81	0.248	6 (3.9)
MSI-H	0.47	0.29, 0.77	**0.003**	81 (53.3)
Surgery & Chemotherapy & Immunotherapy				
MSS	Referent	-	-	4,736 (90.9)
MSI-L	0.85	0.71, 1.03	0.089	200 (3.8)
MSI-H	0.67	0.57, 0.80	**< 0.001**	275 (5.3)
Surgery & Chemotherapy & Radiation				
MSS	Referent	-	-	365 (88.6)
MSI-L	1.00	0.58, 1.73	0.987	22 (5.3)
MSI-H	0.82	0.48, 1.41	0.479	25 (6.1)
All 4				
MSS	Referent	-	-	208 (90.4)
MSI-L	1.64	0.72, 3.73	0.238	8 (3.5)
MSI-H	1.25	0.63, 2.49	0.518	14 (6.1)

## Discussion

Our study demonstrates that MSI status is independently associated with survival outcomes and treatment-associated benefit in colon cancer patients. In this large cohort, 16.8% of tumors were classified as MSI, with the highest prevalence observed in stage II disease. As expected, MSI-high (MSI-H) tumors were more prevalent than MSI-low (MSI-L) tumors, aligning with population-based studies estimating MSI in approximately 9–19% of colorectal cancers [15–17].

MSI status was associated with a distinct clinicopathologic profile. Although MSI tumors are generally linked to more favorable prognostic outcomes, they were more likely to be poorly differentiated compared with MSS tumors, consistent with prior studies demonstrating a strong association between MSI-H status and high-grade histology [9, 16, 18]. MSI tumors were also larger at presentation yet exhibited a lower propensity for metastatic disease [9, 19]. Furthermore, MSI tumors demonstrated a marked predilection for right-sided colon involvement, corroborating well-established anatomic patterns reported in the literature [18, 20, 21].

Most prior studies evaluating the prognostic impact of MSI in colorectal cancer have analyzed colon and rectal tumors together, consistently demonstrating MSI as a favorable prognostic factor in colorectal cancer overall [6, 16, 22, 23]. However, several studies have suggested that the prognostic significance of MSI may differ by tumor site, with evidence indicating that MSI status is not associated with improved outcomes in rectal cancer [24–26]. Notably, Hong et al. reported that MSI-H status was associated with a survival benefit in colon cancer, an effect that was not observed in rectal cancer [24].

Consistent with these observations, our stage-stratified analysis of colon cancer demonstrated a progressively improved survival associated with MSI-H tumors in stages II through IV, whereas MSI-L tumors were associated with a modest survival benefit limited to stage IV disease. Prior studies examining the prognostic role of MSI specifically in colon cancer remain limited [27–30]. Zhang et al. reported that MSI status was associated with reduced disease-specific mortality in patients with stage II colon cancer [29], while Hestetun et al. demonstrated improved prognosis for MSI tumors in stage II but not stage III colon cancer [30].

Given the distinct biological backgrounds of MSI-H tumors, we performed age-stratified analyses to approximate differences between sporadic and Lynch-associated disease. The survival advantage associated with MSI-H was largely consistent across age groups, particularly in stages III and IV, suggesting that its prognostic effect is preserved irrespective of age. While the effect was less pronounced in younger patients with early-stage disease, this may reflect limited sample size rather than true biological differences. These findings support the robustness of MSI-H as a prognostic marker across diverse patient populations.

Contrary to several real-world studies reporting worse outcomes, we observed a survival advantage for MSI-H tumors in stage IV disease, including among patients who did not receive immunotherapy (Supplementary Table 2) [31–33]. This discrepancy may reflect differences in cohort composition and treatment patterns. Additionally, residual confounding and the larger sample size of our study, which provides greater statistical power, may contribute to these findings.

The National Comprehensive Cancer Network (NCCN) recommends routine MSI testing for newly diagnosed colon cancers [34]. While prior studies have compared outcomes across treatment modalities in MSI and MSS tumors, the primary aim of the present study was to evaluate the treatment-modifying effect of MSI status in colon cancer [35–38]. Our analyses demonstrate that in stage I disease, MSI status does not impact prognosis regardless of treatment received, supporting current NCCN recommendations for observation alone in this population [34]. Among patients with stage II colon cancer, MSI-H status was associated with significantly improved survival when treated with surgery alone, an advantage that was not observed in those receiving adjuvant chemotherapy. These findings are consistent with prior evidence suggesting limited benefits of adjuvant chemotherapy in MSI-H stage II tumors and support current ASCO recommendations that reserve adjuvant therapy for select high-risk patients [35, 38–40].

Chemotherapy, administered in the adjuvant or neoadjuvant setting, remains the standard of care for patients with stage III and select stage IV colon cancer, irrespective of MSI status. In this analysis, MSI-H tumors were associated with improved survival among patients treated with surgery and chemotherapy in stage III–IV disease, suggesting persistence of favorable tumor biology in advanced stages. While a survival advantage was also observed among MSI-H patients treated with surgery alone, this finding should be interpreted cautiously and likely reflects favorable tumor biology and selection of patients with limited disease burden rather than a true treatment effect.

MSI tumors have garnered increasing attention in the context of immunotherapy due to their highly immunogenic tumor microenvironment. Le et al. were among the first to establish MSI status as a predictive biomarker of immunotherapy efficacy [13]. More recently, the ATOMIC trial showed that the addition of atezolizumab to mFOLFOX6 improved disease-free survival in patients with stage III dMMR colon cancer [41]. In parallel, multiple studies have demonstrated improved overall survival with immunotherapy in metastatic MSI colorectal cancer [42, 43]. Consistent with these data, our findings support a predictive role of MSI status in immunotherapy-associated survival benefit in stage III–IV colon cancer.

The clinical relevance of MSI-L status remains limited, as MSI-L tumors are widely considered biologically similar to MSS CRCs [44, 45]. In our study, MSI-L status was not consistently associated with survival across treatment groups, with an isolated association observed only among stage IV patients treated with surgery alone, likely reflecting selection bias. These findings align with prior reports by Pawlik et al. and Umar et al., which support the clinical and analytical grouping of MSI-L tumors with MSS disease [46, 47].

A key limitation of this study is the lack of incorporation of BRAF mutation status, which is known to interact with MSI and influence prognosis. Although the NCDB captures BRAF mutation data, this variable was only recently introduced and is missing in approximately 90% of cases, precluding its inclusion in our analysis. MSI-H and BRAF mutations are biologically linked through the CpG island methylator phenotype (CIMP) pathway, and the prognostic impact of MSI-H depends in part on BRAF status [48]. Specifically, BRAF-mutant/MSI-H tumors are associated with worse survival, whereas BRAF–wild-type/MSI-H tumors generally have a more favorable prognosis [49, 50]. Therefore, the inability to account for BRAF status may have influenced the observed associations in our study.

This study is limited by its retrospective design and non-randomized treatment allocation, introducing potential confounding by indication and selection bias. Detailed data on treatment regimens, sequencing, duration, and objective response measures were unavailable, precluding direct assessment of therapeutic response, particularly for immunotherapy. Although multivariable adjustment was performed, residual confounding from unmeasured factors such as performance status, metastatic burden, and molecular co-alterations cannot be excluded. Additionally, small sample sizes in certain treatment subgroups may have limited statistical power, though the overall consistency of findings across stages supports the robustness of the results.

In conclusion, MSI status exhibits a stage-dependent role in colon cancer, functioning as a prognostic biomarker in early-stage disease and a predictive marker for immunotherapy-associated survival benefit in advanced stages. These findings underscore the clinical importance of routine MSI testing to guide molecularly informed treatment decisions across disease stages.

## Supplementary Information

Below is the link to the electronic supplementary material.

Supplementary Material 1 (DOCX 28.0 KB)

## Data Availability

The data that support the findings of this study were obtained from the National Cancer Database (NCDB). These data are not publicly available. Access to the NCDB requires submission of a Participant User File (PUF) application to the American College of Surgeons and the Commission on Cancer, along with institutional approval. Researchers interested in accessing the data may apply directly through the NCDB PUF program (https://www.facs.org/quality-programs/cancer/ncdb/puf/).No newly generated datasets were created for this study.
