# Supplementary material for: Prognostic and Treatment-Associated Survival Effects of Microsatellite Instability Across Disease Stages in Colon Cancer: An NCDB Analysis
Source: J Gastrointest Cancer. 2026 May 20;57(1):117. doi: 10.1007/s12029-026-01493-z (PMC13190752; doi:10.1007/s12029-026-01493-z)
Supplement: Supplementary file 1 — Supplementary Material 1 (DOCX 28.0 KB) [file 12029_2026_1493_MOESM1_ESM.docx]

| **Supplementary table 1.** Age-Stratified Association of MSI Status with Overall Survival by Stage | | | | | | |
| --- | --- | --- | --- | --- | --- | --- |
|  | **HR** | **95% CI** | **P value** | **HR** | **95% CI** | **P value** |
|  | < 50 years old | | | ≥ 50 years old | | |
| **Stage 0** | | | | | | |
| MSS | Referent | - | - | Referent | - | - |
| MSI-L | NA | NA | NA | 0.74 | 0.23, 2.36 | 0.607 |
| MSI-H | NA | NA | NA | 2.09 | 0.88, 4.96 | 0.095 |
| **Stage I** | | | | | | |
| MSS | Referent | - | - | Referent | - | - |
| MSI-L | 1.50 | 0.64, 3.48 | 0.348 | 1.07 | 0.92, 1.23 | 0.377 |
| MSI-H | 0.62 | 0.25, 1.54 | 0.300 | 1.09 | 0.97, 1.22 | 0.143 |
| **Stage II** | | | | | | |
| MSS | Referent | - | - | Referent | - | - |
| MSI-L | 0.97 | 0.61, 1.54 | 0.891 | 0.99 | 0.91, 1.09 | 0.872 |
| MSI-H | 0.71 | 0.50, 1.01 | 0.057 | 0.86 | 0.80, 0.92 | **<0.001** |
| **Stage III** | | | | | | |
| MSS | Referent | - | - | Referent | - | - |
| MSI-L | 0.83 | 0.65, 1.05 | 0.124 | 0.95 | 0.87, 1.03 | 0.175 |
| MSI-H | 0.52 | 0.41, 0.65 | **<0.001** | 0.73 | 0.68, 0.78 | **<0.001** |
| **Stage IV** | | | | | | |
| MSS | Referent | - | - | Referent | - | - |
| MSI-L | 0.90 | 0.72, 1.11 | 0.310 | 0.90 | 0.81, 0.99 | **0.030** |
| MSI-H | 0.70 | 0.56, 0.86 | **<0.001** | 0.63 | 0.57, 0.70 | **<0.001** |

| **Supplementary table 2.** Overall survival by MSI Status in metastatic colon cancer stratified by immunotherapy use | | | | |
| --- | --- | --- | --- | --- |
| **Immunotherapy** | **Stage** | **HR** | **95% CI** | **P value** |
| No | MSS | Referent | - | - |
|  | MSI-L | 0.90 | 0.81, 1.01 | 0.061 |
|  | MSI-H | 0.72 | 0.64, 0.80 | **<0.001** |
| Yes | MSS | Referent | - | - |
|  | MSI-L | 0.87 | 0.73, 1.03 | 0.113 |
|  | MSI-H | 0.64 | 0.54, 0.75 | **<0.001** |
